# Mechanisms of Interference in Vibrotactile Working Memory

**DOI:** 10.1371/journal.pone.0022518

**Published:** 2011-07-26

**Authors:** Tyler D. Bancroft, Philip Servos, William E. Hockley

**Affiliations:** Department of Psychology, Wilfrid Laurier University, Waterloo, Ontario, Canada; The University of Western Ontario, Canada

## Abstract

In previous studies of interference in vibrotactile working memory, subjects were presented with an interfering distractor stimulus during the delay period between the target and probe stimuli in a delayed match-to-sample task. The accuracy of same/different decisions indicated feature overwriting was the mechanism of interference. However, the distractor was presented late in the delay period, and the distractor may have interfered with the decision-making process, rather than the maintenance of stored information. The present study varies the timing of distractor onset, (either early, in the middle, or late in the delay period), and demonstrates both overwriting and non-overwriting forms of interference.

## Introduction

Feature overwriting has been proposed as one of the mechanisms for interference in working memory [1], [2]. In feature overwriting accounts of interference, information is assumed to be stored in a finite set of “feature detectors”. Interfering stimuli (distractors) compete with previously stored representations for access to some of these feature detectors, overwriting the stored representation when successful. As such, the total set of feature detectors contain less information about the initial stimulus, reducing task performance.

Previous theoretical accounts of feature overwriting have used abstract models of feature detectors, but Bancroft and Servos [3] provided a neural basis for overwriting by extending the theory to vibrotactile working memory. Vibrotactile working memory tasks usually use the common delayed match-to-sample paradigm. Subjects are presented with a vibrational stimulus (the “target”, usually to the dominant index finger), followed by a delay period, followed by a second vibrational stimulus (the “probe”), and instructed to report whether the frequencies of the two stimuli match (or, in some cases, whether the probe is of a higher or lower frequency than the target).

Vibrotactile working memory is somewhat unusual, in that it is a cognitive process that is better-understood in animal models than in humans. Substantial research has been done on the neural correlates of vibrotactile working memory, most commonly using single-cell recording in monkeys [4], [5]. Single-cell work has identified four regions thought to be critical in vibrotactile working memory. Further, neurons in these regions appear to share a common neural code for the representation of stimulus information: Firing rates appear to be monotonic (increasing or decreasing) functions of vibration frequency, making it relatively straightforward to determine what information is encoded in any given set of neurons [5].

The four cortical regions involved in vibrotactile working memory are primary somatosensory cortex (SI), secondary somatosensory cortex (SII), prefrontal cortex (PFC), and medial premotor cortex (MPC). For the purposes of this paper, we will focus primarily on SII and PFC: SI is thought to be involved only in initial processing of stimuli, while MPC is thought to largely be involved in preparing motor responses. The firing rates of neurons in PFC contain information about stimulus frequency during stimulus presentation, and this representation appears to persist throughout the delay period [6]. Upon presentation of a probe stimulus, firing rates reflect a comparison between the frequencies of the target and probe stimuli, suggesting that PFC is involved in the same/different (or higher/lower) decision-making process. Firing rates in SII contain information about stimulus frequency during stimulus presentation, and also for approximately 400 ms after stimulus offset [7], [8]. When a probe stimulus is presented, firing rates in SII initially reflect the *target* stimulus frequency for approximately 200 ms, followed by activity similar to the stimulus comparison found in PFC neurons. While the relative roles of SII and PFC in decision-making are unclear, comparison activity in PFC precedes that in SII, suggesting that the comparison may begin in PFC and later spread to SII [6].

The common neural code for frequency and relatively small number of cortical regions involved in vibrotactile working memory make it a good model system for testing theories of interference in working memory. If distractor frequency information partially overwrites stored target information, then the result of the decision-making process should reflect a comparison between the probe and the combination of the target and distractor frequencies, rather than the target frequency alone. Bancroft and Servos [3] tested feature overwriting theory by presenting subjects with a distractor stimulus during the delay period between target and probe. Critically, the frequency of the distractor was a function of probe frequency. On trials where the probe and target were of different frequencies, the distractor frequency could either be shifted towards the probe frequency (for example, a target of 18 Hz, a distractor of 21 Hz, and a probe of 22 Hz), or away from the probe frequency (for example, a target of 18 Hz, a distractor of 15 Hz, and a probe of 22 Hz). If the distractor frequency overwrites the target frequency, we would expect more “same” responses on “towards” trials (as the distractor frequency is closer to the probe frequency than is the target frequency), and more “different” responses on “away” trials (as the distractor frequency is farther away from the probe frequency than is the target frequency). Bancroft and Servos found this pattern of results, suggesting that interference in vibrotactile working memory involves overwriting. Further, their results provided a neural basis for feature overwriting.

Notably, however, Bancroft and Servos presented their distractor stimulus such that there was a 350 ms gap between distractor offset and probe onset. As information tends to persist in SII for approximately 400 ms after stimulus onset, it is possible that their results were not due to the distractor being stored in memory, but rather that distractor information is being incorporated into the decision-making process. In other words, the distractor information is not actually stored in PFC, but rather the persisting activity in SII from processing the stimulus is somehow incorporated into the decision-making process. In this case, the effects are not due to interference with the WM storage and maintenance processes, but rather interference with the decision-making process. The present study aims to clarify this issue by varying distractor timing so that we can compare interference effects when the distractor is presented close to the decision-making period, against effects when the distractor is presented early or in the middle of the delay period, (when, presumably, any effects are due to interference with storage processes). Similar to Bancroft and Servos [3], we would expect these effects to manifest as a significant difference between the number of “different” responses presented on towards-shift and away-shift trials. Alternately, it is possible that distractors may not produce an overwriting effect for a given temporal onset. For example, for middle-onset distractors, subjects have had significant time to switch from encoding to maintenance (whereas early distractors may be presented before subjects have done so), and may not be anticipating the switch from maintenance to decision-making (as in the case of late distractors). If so, subjects may be better able to inhibit the processing of distractors presented in the middle of the delay period.

## Methods

### Participants

Thirty-three undergraduate students from Wilfrid Laurier University participated for course credit. All subjects self-identified as right-handed. Two subjects were excluded from analysis due to performance below chance.

### Apparatus and Procedure

Subjects were presented with vibrational stimuli to the right index finger using a magnetomechanical device similar to those used by Graham et al. [9] and Bancroft and Servos [3]. The device was constructed by gluing a nylon screw to a 63 mm diameter speaker cone, and placing the cone within a plastic housing such that the surface of the screw was flush with the top surface of the housing. The nylon screw was 9 mm in diameter and had a smooth, flat surface with a single groove. Subjects lightly placed their finger on the screw, without applying force. The device was driven by WAV files delivered to the speaker, using an IBM-compatible PC running SuperLab 2.0 (San Pedro, CA: Cedrus). To mask any residual sound from the device, subjects were presented with white noise through headphones, and volume was adjusted until subjects reported they did not hear any residual sound.

Subjects engaged in a brief (80 trials) delayed match-to-sample practice session before beginning the experiment. Subjects were presented with two 1000 ms stimuli, separated by an unfilled 1500 ms delay period. Target and probe stimuli were either the same or different and separated by a 4 Hz frequency difference. Subjects made their responses using their left hand. Subjects were instructed to make a “same” response (by pressing the ‘s’ key on the keyboard) if they believed the probe was the same frequency as the target, and a “different” response (by pressing the ‘d’ key) if they believed the probe was a different frequency from the target. Subjects were provided with visual feedback (correct or incorrect response) after each of the first 40 trials during the practice session only.

During the practice session and the actual experiment, all target stimuli (denoted f1) were 18 or 22 Hz. Probe stimuli (denoted f2) were either the same frequency as the target, or were a different frequency, either 4 Hz above or 4 Hz below the target frequency. Distractor stimuli were all either 3 Hz above or 3 Hz below the target frequency. Target and probe stimuli were presented for 1000 ms each. The delay period was 1500 ms. The distractor stimulus was presented for 250 ms, with an onset of either 250 ms into the delay period (the early condition), 625 ms (the middle condition), or 1000 ms (the late condition). Subjects received 168 same-probe trials and 168 different-probe trials, for a total of 336 trials. Trials were presented in random order. Subjects were instructed to press the ‘s’ key to make a “same” response, and the ‘d’ key to make a “different” response. There was a 500 ms delay between subject response and the beginning of the next trial. Subjects received a short break approximately halfway through the experiment.

## Results

Mean correct same and different responses for each distractor onset condition are reported in Table 1.

**Table 1:** Mean proportion of correct responses for each distractor onset condition.

	Same	Different		Net overwriting effect
		Towards	Away	
Early	.68 (.02)	.52 (.02)	.58 (.02)	.06 (.02)
Middle	.74 (.02)	.50 (.03)	.53 (.03)	.03 (.02)
Late	.71 (.02)	.49 (.03)	.55 (.03)	.06 (.02)

A 2 (test type, same vs. different) X 3 (distractor timing, early vs. middle vs. late) repeated-measures ANOVA was performed on correct responses. A significant main effect of test type was found, *F*(1, 30) = 24.931, *MSe* = .059, *p*<.001, *eta* ^2^ = .454, with higher performance on same trials than on different trials. While the main effect of timing was not significant, (*F*<1), the interaction was, *F*(2, 60) = 4.789, *MSe* = .007, *p* = .012, *eta* ^2^ = .138. Paired-sample *t*-tests were used to break down the interaction, and showed that performance was significantly better for the same/middle than the same/late condition (*t*(30) = 2.999, *p* = .005), but marginally worse for the different/middle condition than the different/early condition, *t*(30) = 2.011, *p* = .053. These results can be interpreted as an overall increase in the number of “same” responses, with subjects making significantly more “same” responses on middle-distractor trials than on early trials, *t*(30) = 3.206, *p* = .003. Subjects also made marginally more same responses on late-distractor than early-distractor trials, *t*(30) = 1.799, *p* = .082.

A 3 (distractor timing, early vs. middle vs. late) X 2 (distractor frequency shift, towards probe vs. away) repeated-measures ANOVA was performed on correct responses to different-probe trials in order to test for overwriting effects. An overwriting effect would appear as significantly more correct “different” responses to away-shift than towards-shift distractors on different-probe trials. There was a significant main effect of frequency shift direction, *F*(1, 30) = 20.028, *MSe* = .006, *p*<.001, partial *eta* ^2^ = .400, confirming the existence of an overwriting effect. The main effect of timing approached significance, *F*(2, 58) = 2.213, *MSe* = .010, *p* = .118, partial *eta* ^2^ = .069, suggesting performance was not equal at all distractor timings. The interaction was not significant, *F*(2, 58) = .688, *p* = .507. Planned paired-sample *t*-tests were performed to compare different-towards and different-away performance in order to determine the existence of an overwriting effect. A significant overwriting effect was present with both early (*t*(30) = 3.325, *p* = .002) and late (*t*(30) = 3.184, *p* = .003) distractors, but not with middle distractors (*t*(30) = 1.341, *p* = .190).

## Discussion

A net overwriting effect was present for early and late distractors, consistent with the distractor overwriting the stored target representation, rather than the distractor being incorporated into the decision-making process. Intriguingly, distractor timing had an effect on overall performance, with middle distractors producing significantly better performance on same-probe trials, and slightly worse performance on different-probe trials. This pattern of results can actually be treated as an increase in the number of same responses, (although this increase was not significant), independent of trial type, as more same responses to same probes will give better performance, and more same responses to different probes will give worse performance. Further, subjects did not exhibit a significant overwriting effect with middle distractors. It was established by Bancroft and Servos [3] that subjects have a bias towards making same responses. Vibrotactile frequency discrimination is a challenging task, independent of the memory aspects of the present task [10]. For subjects to make a “different” response, they must be able to discriminate between the stored representation of the target, and the probe stimulus. If they cannot, either due to a weak stored representation of the target, or due to the psychophysical difficulty of the task, they will make a “same” response.

In this case, the increased number of “same” responses to trials containing a middle distractor could be due to a degraded memory trace. Given the lack of an overwriting effect, middle distractors appear able to interfere with performance in the absence of overwriting. Given the limited research into vibrotactile interference, the mechanism of interference is unclear, but attentional processes are likely to be involved. It is well-established that stimulus processing/encoding and working memory maintenance are separate processes that share some common neural resources [11], and there is evidence that maintenance can affect stimulus encoding [12], [13]. If simultaneous maintenance and encoding of vibrotactile stimuli cannot be performed effectively in parallel, we would expect degradation of stored traces (and reduced performance), as well as reduced encoding of the incoming stimulus (in this case, a reduced overwriting effect). It follows that the effects of simultaneous stimulus processing and encoding would be most pronounced on middle distractors. Middle distractors are processed more thoroughly than early or late distractors, as early distractors overlap with activity in SII that is persisting from the processing of the target stimulus (reducing the degree to which the distractor is processed), and persisting activity in SII from late distractors overlaps with activity from the probe stimulus (also reducing the degree to which the distractor is processed). Further, given the overlap of target/early distractor neural activity, subjects may not engage maintenance processes until distractor offset, preventing deleterious effects due to attentional requirements. In the case of late distractors, encoding processes are engaged at the same time as maintenance processes, but neural activity due to late distractors overlaps with the probe, giving a shorter period of encoding/maintenance overlap than for middle distractors.

It may also be possible that attentional resources are required for the inhibition of middle distractors. Evidence in favour of this explanation comes from Hannula et al. [14], who applied TMS to the middle frontal gyrus (MFG), a region involved in inhibiting activity in primary somatosensory cortex. Increasing activity in MFG during the delay period increased behavioural performance, suggesting that baseline activity in sensory cortex (even in the absence of a stimulus) can interfere with performance. Further, Sörös et al. [15] used fMRI to compare neural activity on vibrotactile working memory tasks with and without a distractor during the delay period, and found increased activity in attention-related regions (including MFG). However, it is not clear why the middle distractor would be inhibited, but not the late distractor.

Alternatively, the lack of an overwriting effect for middle distractors could be attributed to lack of statistical power. However, the sample size used in the present experiment (n = 31) is over twice that used by Bancroft and Servos [3] (n = 14), and was also sufficient to find interference effects for early and late distractors. Additionally, the standard errors found for middle distractors are comparable to those for early and late distractors, suggesting that there is a genuine absence of an overwriting effect.

The existence of a non-overwriting method of interference could be tested using methods already in the literature. Romo et al. have provided measures of how many neurons in a given population contain information about a target stimulus [5]. It would appear relatively straightforward to apply these methods to determining how many PFC neurons are encoding stimulus information after distractor presentation, and whether there is a net loss of total stimulus information. Further, a recent ERP study found that stimulus frequency can be determined based on modulation of frontal activity in the beta band, suggesting that it may be possible to develop a similar measure in humans [16]. Human neuroimaging methods are also well-suited for testing attentional load. Increased attentional cost for middle distractors may present as increased activity in frontal and parietal regions known to be involved in attention [15], [17] and working memory encoding [12].

The present study raises the question of the degree to which distractor encoding leads to loss of stored target information. It may be possible for a distractor to be encoded into working memory without any actual loss of stored target information. For example, if the target stimulus is represented in only a portion of all neurons available for storage, the distractor may be encoded in unused neurons. Alternately, the distractor may overwrite some of the neurons which encode the stored target stimulus, but not to such a degree as to degrade the stored stimulus. In these cases, interference is due not to loss of original information, but rather due to the irrelevant distractor information being included in the decision-making process. The question of how multiple stimuli share the PFC neurons used to store target information is complex, and we are presently approaching this problem via both computational and experimental routes.

In the present study, we demonstrate that feature overwriting in vibrotactile memory is due to interference with the stored contents of working memory. Further, we demonstrate an aspect of interference that does not involve overwriting stored representations. The precise mechanism of this effect is unknown, but may very well involve attentional processes. Future research, both in humans and monkeys, will likely prove fruitful in analyzing the contributions of attention to vibrotactile working memory.
